# Cleaner heating policies contribute significantly to health benefits and cost-savings: A case study in Beijing, China

**DOI:** 10.1016/j.isci.2024.110249

**Published:** 2024-06-11

**Authors:** Zhixiong Weng, Zhaomin Dong, Yi Zhao, Meng Xu, Yang Xie, Feng Lu

**Affiliations:** 1 Institute of Circular Economy, Beijing University of Technology, Beijing 100124, China; 2 School of Materials Science and Engineering, Beihang University, Beijing 100191, China; 3 School of Economics and Management, Beihang University, Beijing 100191, China; 4 School of Management, Wuhan Institute of Technology, Wuhan 430205, China; 5 Beijing Municipal Health Commission Information Center, Beijing 100034, China

**Keywords:** Earth sciences, Environmental science, Environmental health, Environmental management

## Abstract

Cleaner heating policies aim to reduce air pollution and may bring about health benefits to individuals. Based on a fixed-effect model focusing on Beijing, this study found that after the onset of air pollution, daily clinic visits, hospitalization days, and hospitalization expenses increased several days after the occurrence of air pollution. These hospitalization changes were observed in males and females and three different age groups. A difference-in-differences (DID) model was constructed to identify the influences of cleaner heating policies on health consequences. The study revealed that the policy positively affects health outcomes, with an average decrease of 3.28 thousand clinic visits for all diseases. The total hospitalization days and expenses tend to decrease by 0.22 thousand days and 0.34 million CNY (Chinese Yuan), respectively. Furthermore, implementing the policy significantly reduced the number of daily clinic visits for respiratory diseases, asthma, stroke, diabetes, and chronic obstructive pulmonary diseases (COPDs).

## Highlights

• Cleaner heating policies lead to a significant decline in hospitalization expenses
• Hospitalization changes caused by cleaner heating policies differ in gender
• Different age groups suffered differences from the cleaner heating policies
• Cleaner heating policies reduced the number of clinic visits for different diseases

## Graphical abstract

**Figure:**
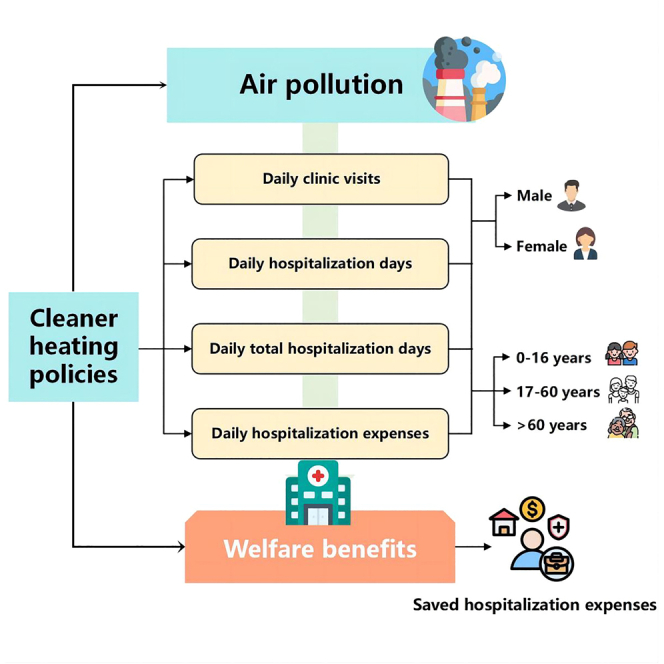

## Introduction

Using household cleaner heating energy is an effective way to reduce carbon emissions and improve air quality. China has implemented a comprehensive cleaner heating policy since 2017 to achieve clean air goals. This policy covers the majority of rural households in the northern part of the country.^1^^,^^2^^,^^3^ Several measures have been taken to reduce the severe air pollution caused by traditional winter heating, such as switching from coal to natural gas or electricity.^4^^,^^5^^,^^6^^,^^7^ In China, over 37 million rural households have successfully transitioned to cleaner heating by late 2022 under the pressure of top-down directives.

In 2017, the “Beijing-Tianjin-Hebei 2017–2018 Autumn and Winter Comprehensive Air Pollution Control Action” was implemented, initiating cleaner heating policies. This plan identified the task of transforming heating systems to cleaner alternatives in 28 cities in Beijing-Tianjin-Hebei and surrounding areas (“2 + 26” cities). The deadline for completion was October 2017. To ensure successful completion, strict coal-to-natural gas and coal-to-electricity heating transformation tasks were adopted. Large-scale cleaner heating retrofitting continued in 2018 and 2019. By the end of 2022, a total of 37 million households in northern rural areas of China have been renovated for cleaner heating.

The cleaner heating policies have several distinct features. Firstly, the policies adopt a top-down task decomposition model, whereby the Ministry of Ecology and Environment, responsible for ecological matters, assigns cleaner heating transformation tasks to various regions yearly. These tasks are then decomposed step by step to the provinces, prefecture-level cities, counties, towns, and villages, creating a clear responsibility decomposition system. Secondly, the implementation of cleaner heating policies is extensive, encompassing most rural areas in northern China in addition to the “2 + 26” cities. This policy involves different levels of government, farmers, heating and gas suppliers, and infrastructure transformation enterprises. Thirdly, the central and local governments offered significant financial subsidies through economic incentive policies to support cleaner heating renovation in different regions in the first few years of the policy’s implementation. Overall, the cleaner heating policies’ features align with top-down task decomposition models, extensive implementation, and financial incentives, demonstrating a concerted effort to promote the implementation of cleaner heating policies in China.

The city of Beijing has implemented several stages of cleaner heating policies, with the most rigorous implementation occurring at the beginning of 2017. The objective was to meet the “Air Pollution Prevention and Control Action Plan” assessment goals within the final year. In order to achieve this, Beijing has significantly increased the implementation of cleaner heating, resulting in a large-scale transition from coal to natural gas and electricity, with an emphasis on coal to electricity. Following several years of transformation efforts, Beijing has largely completed the cleaner heating task. As a consequence, millions of rural households have undergone cleaner heating transformations, enabling cleaner winter heating on the household side between 2017 and 2022.^8^

Implementing strict measures for a cleaner heating policy has been proven to significantly improve air quality.^9^^,^^10^^,^^11^^,^^12^ Researchers have revealed that the implementation of cleaner heating policies resulted in a decrease of air pollutants such as PM_2.5_ and PM_10_ by 3.4 μg/m^3^ and 5.3 μg/m^3^, respectively, in the “2 + 26” cities.^13^ Previous studies have identified that subsidizing electric heat pumps and electricity in Beijing’s rural regions eliminated coal use resulting in benefits to indoor air pollution.^14^ These reduction effects were also observed in other provinces, including Shandong, which experienced a decline of 7.32% in PM_2.5_ following the implementation of cleaner heating policies.^15^ The implementation of cleaner heating policies is expected to result in a reduction in air pollution, which in turn is likely to contribute significantly to the improvement of individuals’ health. This positive correlation between air quality enhancements and the prevention of various diseases stems from the fact that fresh air contains more oxygen, which improves lung function and strengthens the body’s immunity, thereby reducing the incidence of respiratory diseases. Additionally, the improved air quality positively impacts cardiovascular health by decreasing the damage that inhaling harmful substances causes to the cardiovascular system.

In contrast to the previous studies that have measured the environmental benefits brought about by cleaner heating policies,^2^^,^^16^^,^^17^^,^^18^ the scientific new contributions of our study can be concluded as follows:

Firstly, our study clearly revealed the health benefits of cleaner heating policies. Prior studies have primarily focused on assessing the health advantages for individuals resulting from improvements in air quality,^19^^,^^20^^,^^21^^,^^22^^,^^23^ but they did not determine the health benefits specifically to the cleaner heating policies. Our research has shown that the adoption of cleaner heating policies resulted in a noteworthy decrease in clinic visits for all kinds of diseases. This provides a fresh outlook on evaluating the health advantages that the implementation of cleaner heating policies can offer.

Secondly, our study precisely measured the health-related expenses related to cleaner heating. We conducted a study on daily health expenses in Chinese cities to investigate whether people’s medical treatment habits change in response to improvements in air quality resulting from cleaner heating policies. Our research sheds light on the medical cost savings associated with implementing cleaner heating policies and quantifies the benefits of reducing the burden of medical expenses.

Thirdly, our study provided measurements on the potential welfare gains of implementing cleaner heating policies. We measured the cost savings in healthcare expenditures at the city level as a result of implementing cleaner heating policies. Our findings suggest that continuing to implement such policies can lead to significant savings in hospitalization expenses. This information is useful for policymakers, as it highlights the economic benefits of promoting environmental policies.

Our study aimed to determine the link between air pollution and health outcomes in Beijing’s 16 districts (Various variables can be seen in Table 1). Using a difference-in-differences model (DID), we analyzed the daily impact of cleaner heating policies on clinic visits, hospitalization days, and healthcare expenses in Beijing, which indicates a health-related behavioral variation due to the implementation of cleaner heating policies. Our study also provided comprehensive measurements of the saved hospitalization expenses in Beijing, implying numerous welfare benefits to individuals by continually implementing cleaner heating policies in the long run.

**Table 1:** Summary statistics

Variables	Variable description	Unit	Obs.	Mean	Min	Max
all_hosp_tot_male	Hospitalization days of males for all diseases	days	29,216	1.92 thousand	12	88.16 thousand
all_hosp_tot_female	Hospitalization days of females for all diseases	days	29,216	1.81 thousand	27	105.78 thousand
all_hosp_tot_016y	Hospitalization days of patients aged 0–16 for all diseases	days	29,216	0.12 thousand	0	7.74 thousand
all_hosp_tot_1760y	Hospitalization days of patients aged 17–60 for all diseases	days	29,216	1.94 thousand	29	103.13 thousand
all_hosp_tot_larg60y	Hospitalization days of patients aged more than 60 for all diseases	days	29,216	1.97 thousand	19	17.35 thousand
all_treatexp_male	Hospitalization expenses of males for all diseases	Yuan	29,216	3.43 million	0.32 million	24.30 million
all_treatexp_female	Hospitalization expenses of females for all diseases	Yuan	29,216	3.10 million	0.04 million	23.90 million
all_treatexp_016y	Hospitalization expenses of patients aged 0–16 for all diseases	Yuan	29,216	0.19 million	0	2.38 million
all_treatexp_1760y	Hospitalization expenses of patients aged 17–60 for all diseases	Yuan	29,216	2.63 million	0.04 million	18.50 million
all_treatexp_larg60y	Hospitalization expenses for patients more than 60 for all diseases	Yuan	29,216	3.69 million	0.02 million	27.80 million
avgtemp	Daily average temperature	°C	29,216	13	−21	33
maxtemp	Daily maximum temperature	°C	29,216	18	−20	40
mintemp	Daily minimum temperature	°C	29,216	8	−24	29
wingrd	Daily wind grades	–	29,216	2	0	8
precip	Daily precipitation	mm	29,216	0.33	0	62
press	Daily barometric pressure	Pa	29,216	99.55 thousand	87.78 thousand	103.78 thousand
pm25	Daily PM_2.5_ concentration	μg/m^3^	29,216	130	4	534
pm10	Daily PM_10_ concentration	μg/m^3^	29,216	183	6	734

Specifically, we used a fixed-effect model to estimate the causal relationship between air pollutants, primarily PM_2.5_ and PM_10_ concentrations, and health-related conditions in Beijing. We analyzed clinic visits, hospitalization days, and hospitalization expenses data at the district level to measure this relationship. We comprehensively considered the influence of air pollutants on hospitalization behaviors with a lag of 0–7 days to capture air pollution’s characteristics. Next, we constructed a DID model to determine if the cleaner heating policies resulted in a decline in districts’ hospitalization behaviors and related healthcare expenditures. We further investigated the policies’ heterogeneous effects on males and females, as well as on three age groups (0–16, 17–60, and more than 60 years old) in all districts of Beijing as a heterogeneity test. Lastly, we measured each district’s economic benefits in Beijing based on the implementation of consecutive cleaner heating policies from 2017 to 2022.

## Results

### Health-related consequences caused by air pollutants

**Figure 1:**
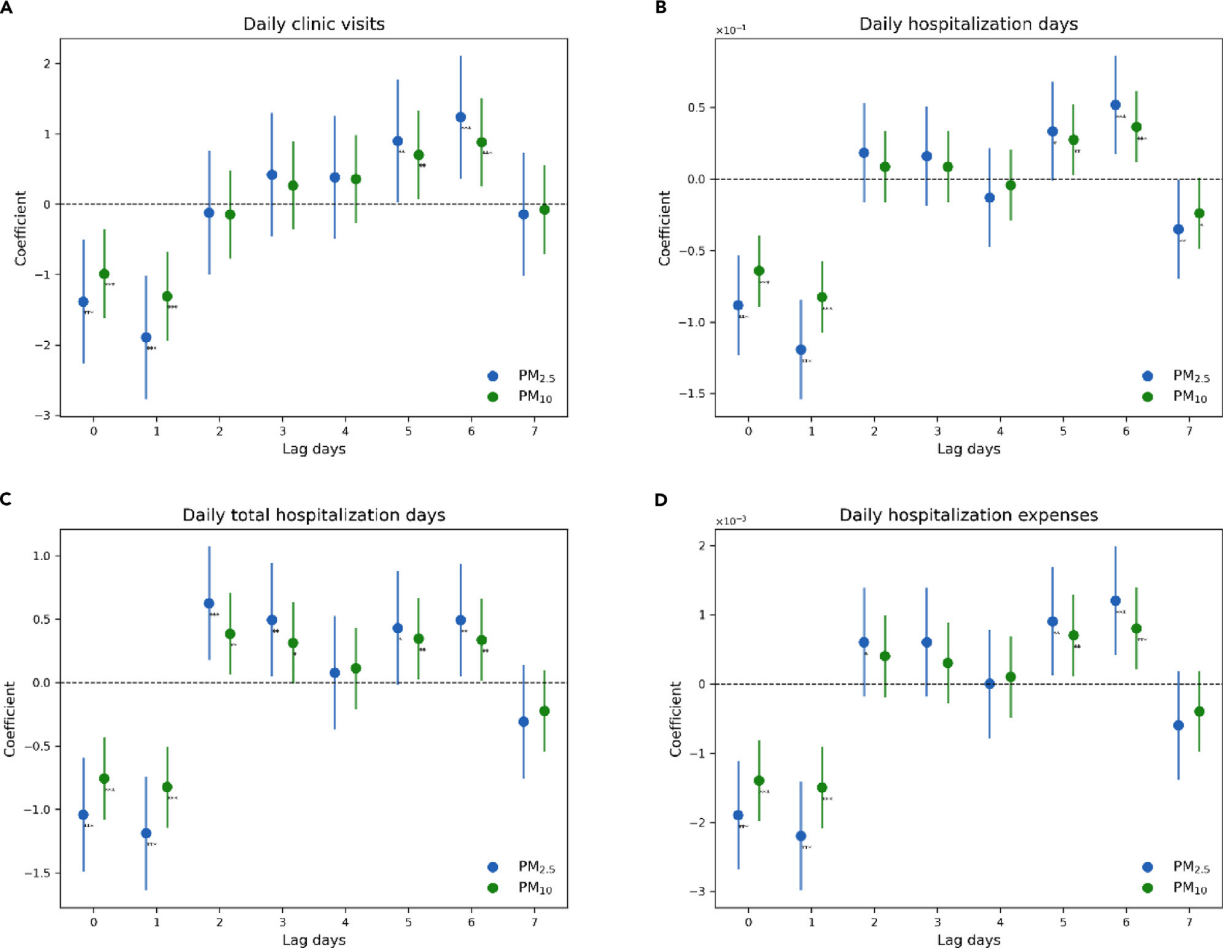
Effects of air pollutants on hospitalization-related consequences (A) Effects on daily clinic visits. (B) Effects on daily hospitalization days. (C) Effects on daily total hospitalization days. (D) Effects on daily hospitalization expenses.

To assess the effects of pollutants on health, we monitored the concentrations of PM_2.5_ and PM_10_ with a lag time ranging from 0 to 7 days (Figure 1). This is because the concentration of air pollutants in the human body accumulates over time, and within a week, the impact on the human body becomes more profound. In our study, for instance, December 7th was considered the 0 days, December 6th was the lagged 1 day, December 5th was the lagged 2 days, and so on. This allowed us to identify the impact of pollutant concentrations on health under different lag times. There is a significant increase in daily clinic visits after a delay of 3–6 days caused by air pollution (Figure 1A). For instance, clinic visits started to increase on the third day following the onset of air pollution, and this trend continued until the fifth and sixth days. Specifically, on the fifth and sixth days of being exposed to air pollutants (PM_2.5_ and PM_10_), the clinic visits increased by 0.9 and 1.2, respectively, for each additional unit of PM_2.5_ concentration. Similarly, when taking PM_10_ concentrations into account, the results of the increasing number of clinic visits were similar, at 0.7 and 0.9, respectively, on the fifth and sixth days of being exposed to PM_2.5_ and PM_10_. On the fifth and sixth days following exposure to these air pollutants, there was a statistically significant increase in the number of days patients had to stay in the hospital. For example, on the sixth day after being exposed to these air pollutants (Figure 1B), hospitalization days increased by an additional 0.05 and 0.04 days for each one-unit increase of PM_2.5_ and PM_10_ concentration, respectively.

Patients who were exposed to air pollution had larger daily total hospitalization days. Exposure to air pollutants led to an increase in total hospitalization days, but the effects were delayed by two days and lasted for approximately four days (Figure 1C). For instance, the number of days people spent in the hospital increased by 0.6 and 0.4 on the second day of being exposed to air pollutants (PM_2.5_ and PM_10_), respectively. This increase was sustained until the sixth day, after which the total hospitalization days increased by 0.5 and 0.3 for each additional unit of PM_2.5_ and PM_10_ concentration, respectively.

Our study found a positive correlation between air pollutants, specifically PM_2.5_ and PM_10_, and daily hospitalization expenses. We discovered that an increase in clinic visits and hospitalization days leads to higher hospitalization expenses. This correlation was particularly significant on the fifth and sixth days following the exposure to air pollutants. On the fifth day of being exposed to air pollutants (Figure 1D), an increase of one additional unit in PM_2.5_ and PM_10_ concentrations resulted in an increase of 0.0009 and 0.0007 million CNY, respectively.

### Hospitalization consequences across gender and age

**Figure 2:**
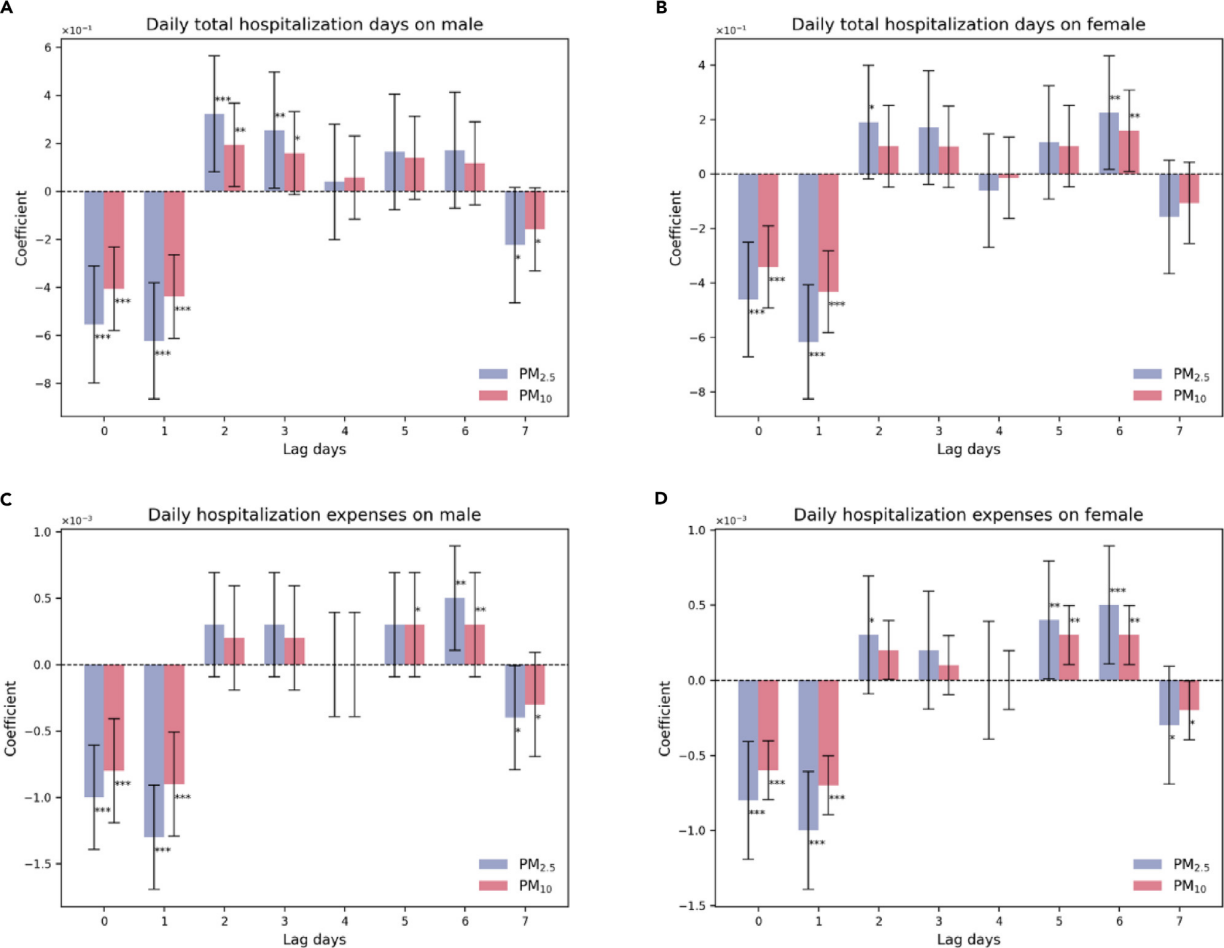
Effects of air pollutants on hospitalization-related consequences by gender (A) Effects on daily hospitalization days on male. (B) Effects on daily total hospitalization days on female. (C) Effects on daily hospitalization expenses on male. (D) Effects on daily hospitalization expenses on female.

We have analyzed the impacts of lagged air pollutants on the total hospitalization days and hospitalization expenses for both males and females. Our study reveals that air pollutants have a significant impact on the total hospitalization days of both genders. We observed that the total hospitalization days for males started to increase on the second and third days of air pollution formation (Figure 2A). This implies that for every additional increase of PM_2.5_ and PM_10_ concentration, there was a corresponding increase in males’ total hospitalization days by 0.3 and 0.2, respectively. However, as the lag days increased, the impact of the lagged air pollutants on the total hospitalization days decreased. After the fourth day of being exposed to air pollution, there was no statistically significant effect on the total hospitalization days. On the other hand (Figure 2B), the increasing effect on the total hospitalization days for females occurred on the sixth day following the exposure to air pollutants. This suggests that for every additional unit increase of PM_2.5_ and PM_10_ concentration, there was a corresponding increase in total hospitalization days by 0.2 and 0.2, respectively.

Hospitalization expenses tend to increase with the rise in air pollution levels for both males and females. This relationship was observed in both males and females (Figures 2C and 2D). Additionally, we discovered that air pollution has a delayed effect on hospitalization expenses. For instance, an increase in PM_10_ concentration by one unit caused daily hospitalization expenses for males and females to increase by 0.0005 million CNY each on the sixth day of being exposed to PM_2.5_ pollution. Similarly, when measured by the PM_10_ influence, males’ and females’ daily hospitalization expenses increased by 0.0003 million CNY each on the fifth day after being exposed to air pollution.

**Figure 3:**
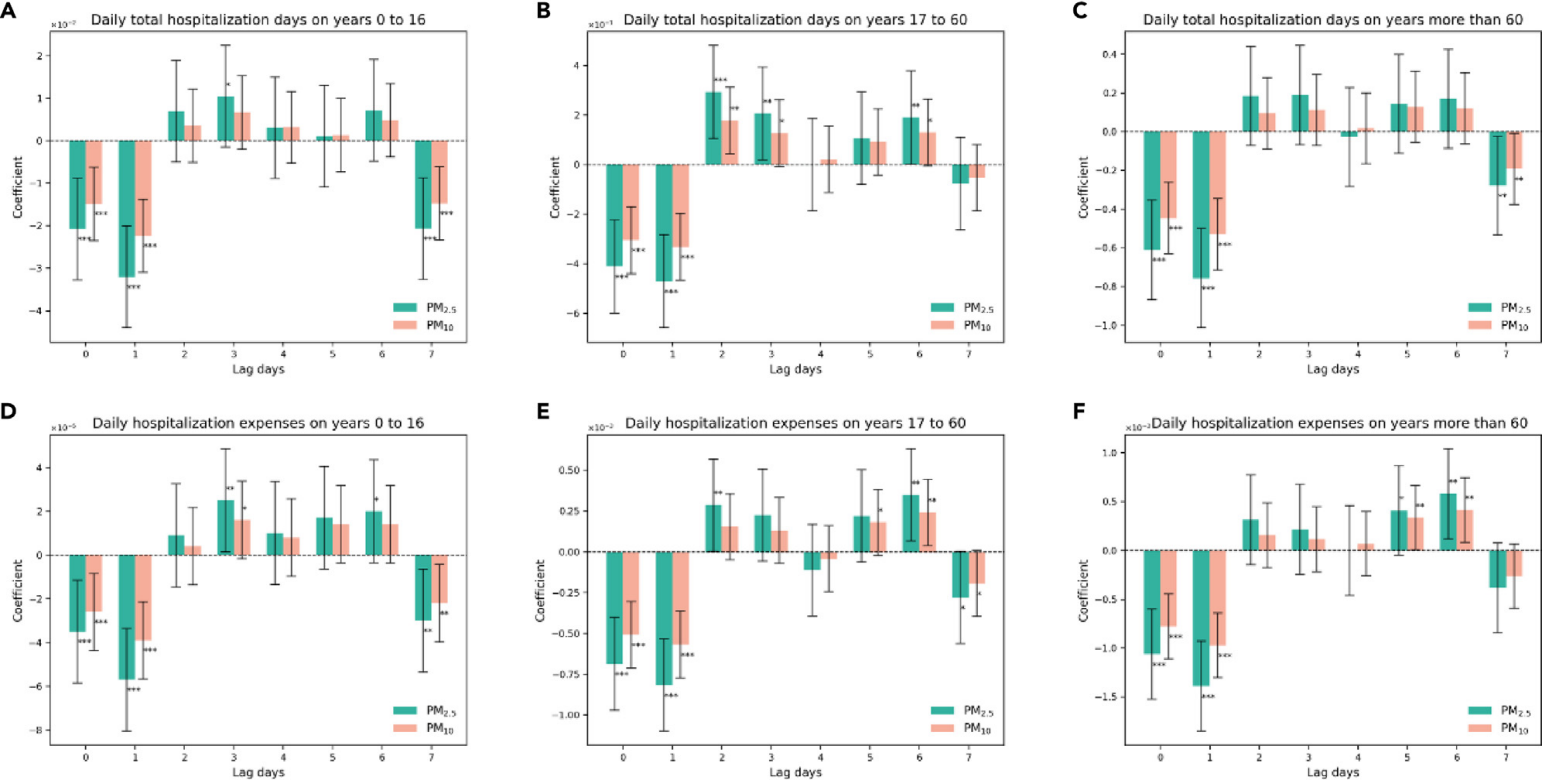
Effects of air pollutants on daily total hospitalization days and hospitalization expenses by age groups (A) Effects on daily total hospitalization days of years 0–16. (B) Effects on daily hospitalization days of years 17–60. (C) Effects on daily hospitalization days on years more than 60. (D) Effects on daily hospitalization expenses of years 0–16. (E) Effects on daily hospitalization expenses of years 17–60. (F) Effects on daily hospitalization expenses of years more than 60.

We estimated the impact of air pollutants on the total number of hospitalization days for three different age groups (Figure 3A–3C). The study shows that air pollutants have a significant lagged effect on the daily total hospitalization days for the age group between 17 and 60 years old but not for the other two age groups. Specifically, an additional unit increase of PM_2.5_ concentration with a 2-day lag led to a 0.3 increase in daily total hospitalization days for the 17–60 age group. On the other hand, taking PM_10_ concentrations into account resulted in a daily hospitalization day increase of 0.2 for the same age group. On the sixth day of being exposed to air pollutants (PM_2.5_ and PM_10_), the 17–60 age group experienced a 0.2 and 0.1 increase in daily hospitalization days, respectively.

All three age groups experienced a rise in hospitalization expenses due to the delayed effects of air pollution (Figure 3D–3F). The hospitalization expenses increased significantly two or three days after being exposed to air pollutants and continued for several days. For instance, the daily hospitalization expenses for the 0–16 age group increased by 0.000025 million CNY on the second day of being exposed to PM_2.5_, implying that an increase in the concentration of PM_2.5_ resulted in substantial additional health-related costs. Similarly, the 17–60 age group incurred an extra cost of 0.000285 million CNY with a 2-day lag in PM_2.5_ pollution. Moreover, this increasing trend persisted for several days. The hospitalization expenses for the 17–60 and over 60 age groups rose by 0.000348 and 0.000580 million CNY, respectively, with a 6-day lag of being exposed to PM_2.5_.

It is worth noting that all sub-figures in both Figures 2 and 3 displayed the same pattern. In the presence of air pollution on the first day and the following day, all health-related outcomes decreased. For instance, the daily hospitalization expenses for males decreased by 0.001 and 0.0013 million CNY on the day when PM_2.5_ pollution occurred and the next day (Figure 2C), respectively. Different age groups also exhibited a similar trend. Hospitalization expenses for the age group of 17–60 decreased by 0.000687 and 0.000817 million CNY on the day when being exposed to PM_2.5_ on the first day and the next day (Figure 3E), respectively. This is because air pollution can have a significant impact on people’s health. To avoid exposure to air pollution, people often prefer to stay indoors. This may help reduce clinic visits, hospitalization days, and expenses in the short term. However, the concentration of air pollutants increases over time, leading to physical discomfort after a few days. As a result, there may be an increase in clinic visits and hospitalization days on the fifth and sixth days of exposure to air pollution. Ultimately, this can lead to higher hospitalization expenses.

### Cleaner heating policy on hospitalization behavior variation

The implementation of a cleaner heating policy in Beijing significantly impacts hospitalization rates (panel A in Table 2). Health outcomes are positively affected by the policy, with an average decrease of 3,280 clinic visits for all diseases. Additionally, total hospitalization days and expenses tend to decrease by 218.96 days and 0.34 million CNY, respectively.

Panel B in Table 2 shows that implementing the cleaner heating policies led to a significant reduction in the number of daily clinic visits for respiratory diseases, asthma, stroke, diabetes, and chronic obstructive pulmonary diseases (COPD). On average, the number of daily clinic visits for these diseases decreased by 49.99, 0.06, 0.45, 10.10, and 60.16, respectively.

**Table 2:** Effect of cleaner heating policies on health outcomes

Panel A: Clean heating policy on daily health-related outcomes (All diseases)				
	Clinic visits	Hospitalization days	Total hospitalization days	Hospitalization expenses
cleanheat_post	−3,280∗∗∗ (151.2993)	−6.90 (6.2957)	−218.96∗∗∗ (82.6756)	−0.34∗∗ (0.1345)
N	9,696	9,696	9,696	9,696
adj. R^2^	0.705	0.623	0.540	0.617

Panel B: Cleaner heating policy on different diseases’ daily clinic visits					
	Respiratory	Asthma	Stroke	COPD	Diabetes
cleanheat_post	−49.99∗∗∗ (2.7159)	−0.06∗∗∗ (0.0073)	−0.45∗∗∗ (0.0539)	−10.10∗∗∗ (0.4616)	−60.16∗∗∗ (3.9043)
N	9,696	9,696	9,696	9,696	9,696
adj. R^2^	0.769	0.414	0.458	0.705	0.649

Panel C: Cleaner heating policy on genders and age groups (Daily total hospitalization days for all diseases)					
	Male	Female	0-16 years old	17-60 years old	>60 years old
cleanheat_post	−117.04∗∗∗ (44.2255)	−75.10∗ (38.5986)	−5.24∗∗ (2.2041)	−69.05∗ (35.2956)	−105.89∗∗ (46.2523)
N	9,696	9,696	9,696	9,696	9,696
adj. R^2^	0.492	0.574	0.625	0.500	0.560

Panel D: Cleaner heating policy on genders and age groups (Daily hospitalization expenses for all diseases)					
	Male	Female	0-16 years old	17-60 years old	>60 years old
cleanheat_post	−0.18∗∗ (0.0743)	−0.16∗∗∗ (0.0621)	−0.01∗∗∗ (0.0043)	−0.01 (0.0515)	−0.32∗∗∗ (0.0826)
n	9,696	9,696	9,696	9,696	9,696
adj. R^2^	0.581	0.643	0.624	0.596	0.619

Notes: Standard errors are in parentheses. Significance: ∗∗∗ 1%, ∗∗ 5%, and ∗ 10%.

In contrast, panel C of the same table reflects the effect of the cleaner heating policy on the number of daily hospitalization days for all diseases across different age groups and genders. Both males and females were found to be significantly affected by the cleaner heating policy, with no evident differences in policy effects. More specifically, the number of daily hospitalization days for all diseases for males and females decreased by 117.04 and 75.10 days, respectively, after the implementation of the cleaner heating policy.

Additionally, all three age groups (0–16, 17–60, and more than 60 years old) were found to have reduced their daily hospitalization days due to the cleaner heating policy. The older age group (more than 60 years old) exhibited the largest reduction in hospitalization days, with a decrease of 105.89 days, compared to the age groups of 0–16 (5.24 days) and 17–60 (69.05 days).

In panel D, it is shown that the cleaner heating policy has a significant impact on daily hospitalization expenses, categorized by gender and age groups. Both males and females experienced a reduction in daily hospitalization expenses for all diseases, with a reduction magnitude of 0.18 million CNY and 0.16 million CNY, respectively. Moreover, the implementation of the cleaner heating policies resulted in greater benefits for the young (0–16 years old) and old age groups (more than 60 years old), with a decrease of 0.01 million CNY and 0.32 million CNY in daily hospitalization expenses for all diseases, respectively.

### Welfare benefits brought by cleaner heating policies

**Figure 4:**
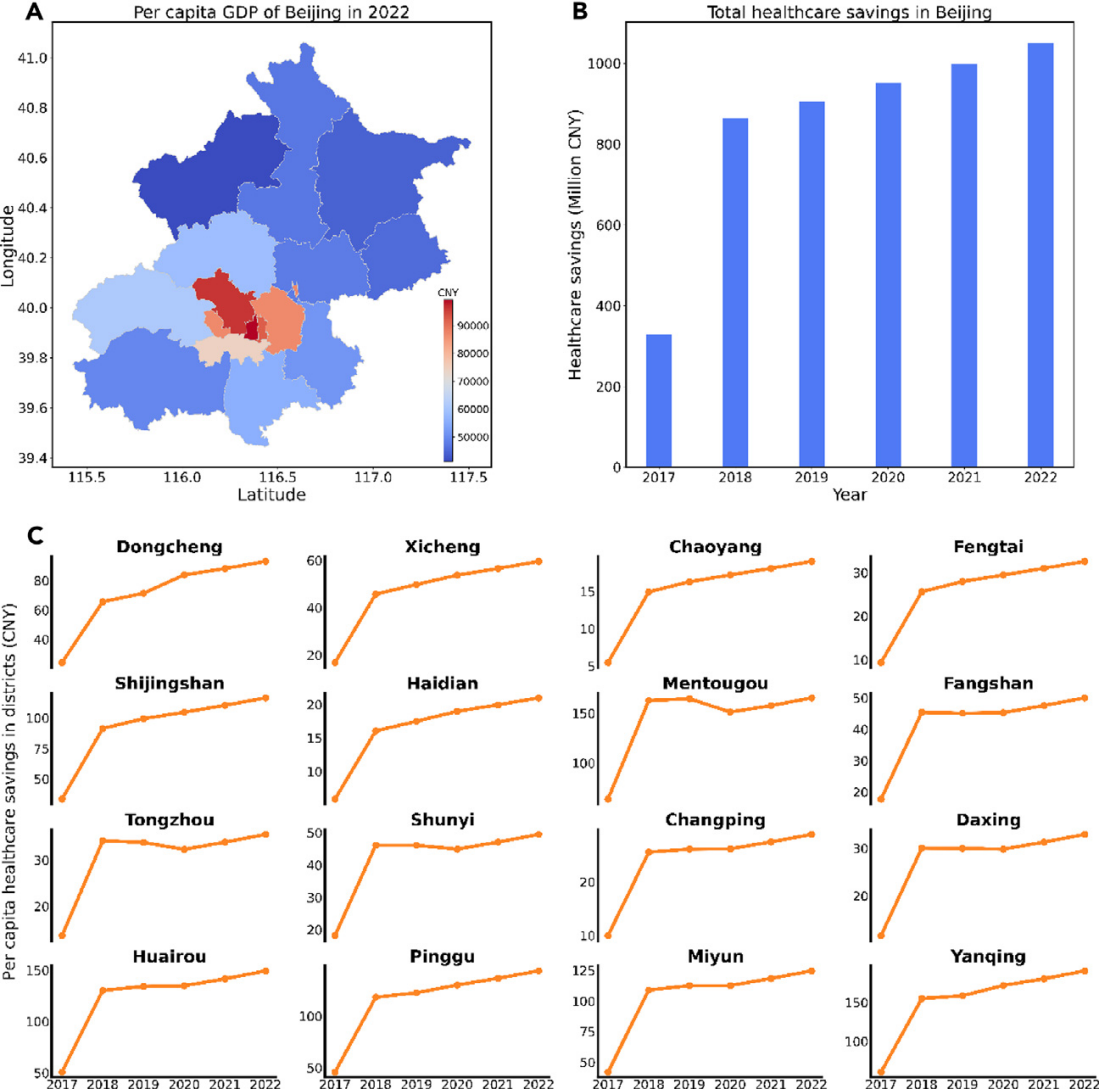
Potential healthcare savings brought by the cleaner heating policies in Beijing (A) The per capita GDP (unit: CNY) of each district in Beijing in 2022. (B) The total healthcare savings (unit: Million CNY) brought by the cleaner heating policies from 2017 to 2022. (C) The per capita healthcare savings (unit: CNY) for each district resulting from the cleaner heating policies between 2017 and 2022).

Figure 4 illustrates the potential welfare benefits for the districts in Beijing from 2017 to 2022 when the cleaner heating policies were implemented. The policy was assumed to have an equal impact on all the districts and resulted in a decrease of 0.3426 million CNY in daily hospitalization expenses in the base year. Each year since 2017, cleaner heating policies have been increasingly implemented in Beijing. To assess the policies’ welfare effect on reducing hospitalization expenditures from 2017 to 2022, we assumed a 5% yearly policy improvement effect. Overall, the cleaner heating policies have significantly reduced hospitalization expenses (see Figure 4B), amounting to around 328.90 million CNY in 2017 and 1,049.41 million CNY in 2022. Additionally, the cleaner heating policies have saved approximately 5,099.46 million CNY in hospitalization expenses in Beijing between 2017 and 2022.

We have measured the benefits of the cleaner heating policies in terms of per capita hospitalization expenses for each district in Beijing. The implementation of the policies has resulted in significant benefits for all districts in Beijing (see Figure 4C). For instance, the Xicheng district, which is the most economically prosperous district in Beijing (see Figure 4A), has received an average per capita hospitalization expense benefit of 24.2 CNY in 2017 and 93.2 CNY in 2022. Due to the size of the population, districts with smaller populations have received greater savings in per capita hospitalization expenses. Our study found that the Yanqing district had the highest expense benefits, with a per capita hospitalization expense of 190.7 CNY in 2022. Similarly, the Mentougou, Huairou, Pinggu, and Miyun districts have relatively high benefits, with 165.6, 149.4, 143.8, and 124.7 CNY in 2022, respectively. Moreover, all districts have shown an increasing trend in the benefits of per capita hospitalization expenses as a result of the cleaner heating policies.

## Discussion

According to our analysis, air pollutants such as PM_2.5_ and PM_10_ have significant delayed effects on hospitalization behavior and expenses in Beijing. After the onset of air pollution, clinic visits increased on the third day and continued until the fifth and sixth days. On the fifth- and sixth-days following exposure to these pollutants, there was a statistically significant increase in the number of days patients had to stay in the hospital. Patients who stayed in the hospital for longer periods faced higher hospitalization expenses. Our findings show that on the fifth day after the occurrence of air pollution, an increase of one additional unit in PM_2.5_ and PM_10_ concentrations resulted in an increase of 0.0009 and 0.0007 million CNY, respectively. Studies conducted on the effects of air pollution on healthcare have consistently found that exposure to PM_2.5_ is a significant contributing factor to premature deaths,^24^^,^^25^^,^^26^ all-cause mortality risk,^27^^,^^28^ and a range of cause-specific diseases.^29^^,^^30^^,^^31^ The negative impact of air pollution on human health is not immediate and tends to increase with prolonged exposure, resulting in a delayed impact.^32^^,^^33^ Therefore, as air pollution levels increase, related health expenditures are likely to rise as well.^34^^,^^35^^,^^36^ These findings are consistent with previous research on the subject.

According to our research, air pollution can have varying impacts on health, depending on the gender and age of individuals. Our findings indicate that both men and women are likely to experience increased clinic visits, hospitalization days, and hospitalization expenses as air pollution levels rise. Some investigations have suggested that males may be more susceptible to the detrimental effects of atmospheric pollution.^37^ However, the primary reason for this is that men tend to undertake work in outdoor settings, and hence, are more exposed to atmospheric pollution, rendering them more vulnerable to health implications. We have not found any evidence to support the previously mentioned conclusions. This is mainly due to two reasons. Firstly, a higher number of women opt to work in Beijing due to the abundance of job opportunities available, which increases the likelihood of both men and women being exposed to air pollution. Secondly, the available healthcare records in our study do not provide any distinction based on the job categories of individuals. Instead, they are solely focused on presenting the health outcomes at the district level. As a result, there are insignificant differences in the influence of gender. Additionally, three age groups (namely 0–16, 17–60, and more than 60 years old) exhibit similar trends in health-related behaviors and expenditures due to the delayed effects of air pollution, indicating that daily clinic visits, hospitalization days, and hospitalization expenses increase several days after the occurrence of air pollution.

We conducted an evaluation to determine how effective the cleaner heating policies were in reducing healthcare and hospitalization expenses in Beijing. Our study findings indicate that the adoption of the aforementioned policies has resulted in a significant reduction in daily clinic visits, overall hospitalization days, and associated costs. Notably, the policies have proven to be particularly efficacious in curbing clinic visits for five distinct ailments, which include respiratory, asthma, stroke, COPD, and diabetes. Moreover, these policies led to a reduction in hospitalization days for all age groups and both genders, ultimately resulting in a significant decrease in hospitalization expenses. It has been observed that implementing stringent measures to control air pollution can have a positive impact on reducing healthcare expenses. Studies have shown that the benefits reaped from cleaner heating policies were comparable to the health benefits achieved by China’s clean air actions. Therefore, it can be inferred that taking steps to reduce air pollution can not only improve the quality of air but also have a positive impact on public health and healthcare expenses.^36^^,^^38^ Furthermore, we measured the potential welfare brought about by the cleaner heating policies for each district in Beijing. Similar to other studies,^39^^,^^40^ economic welfare benefits in air pollution control policies were found to be considerable.

Our findings suggested that the implementation of cleaner heating policies can result in a significant reduction in hospitalizations that are associated with air pollution at the district level. This information is of utmost importance to policymakers who are seeking to mitigate healthcare expenses. To minimize air pollution during the winter season, it is recommended that developing countries prioritize transitioning from coal to natural gas or electricity for heating purposes. It is imperative that regional economic disparities are taken into account and economic incentives, such as fiscal transfers, are provided to poorer regions to support the implementation of cleaner heating policies. Policymakers should consider the long-term economic sustainability of their policies and ensure that the policies adopted are financially viable in the long run.

### Limitations of the study

Some limitations must be acknowledged. Firstly, we mainly focused on hospitalization-related changes at the district level, as we did not have access to household or individual-level data. This means we were unable to analyze the characteristics of the individuals affected by the policies. Additionally, the lack of analysis on the individuals may also lead to an uncompleted control for the potential influencers that may affect health-related outcomes. Secondly, we did not provide sufficient analysis of the influence of policies on different diseases. This is because there were insufficient numbers of cases to provide the power needed to do a disease-specific analysis. Thirdly, we conducted a welfare analysis of cleaner heating policies at the district level, but we were not able to measure their economic burdens if implemented over a longer period.

## STAR★Methods

### Key resources table

**Table:** 

REAGENT or RESOURCE	SOURCE	IDENTIFIER
**Deposited data**		

County-level health-related data	Beijing Municipal Health Commission	RRID: AB_2313773
Daily air pollutant concentration data	China National Urban Air Quality Real-time Publishing Platform	https://air.cnemc.cn:18007/

**Software and algorithms**		

STATA V.15	STATA Version 15	http://www/stata.com/stata15/

### Resource availability

#### Lead contact

All requests regarding data and codes and clarification of the manuscript should be directed to the corresponding author Yang Xie (xieyangdaisy@buaa.edu.cn).

#### Materials availability

The manuscript did not employ any physical material. It is a pure data analysis work.

#### Data and code availability

• The health-related data used in this manuscript has a legal host. Interested readers have to contact the hosting institution for the data. Other datasets are available with access to the listed websites in the key resources table.
• The codes used to obtain the results in this study can be made available to interested readers upon reasonable request from the corresponding authors.
• Further clarification and information on the manuscript and data should be addressed to the corresponding authors with the coauthors in copy.

### Method details

#### Fixed-effect model

We have used a fixed-effect model to analyze the causal relationship between air pollutants and hospitalization patterns and expenses. To account for the specific transmission path and lag effect of air pollutants on the population, we have included the lagged characteristics of air pollutants in the model. The basic specifications of the fixed-effect model were established as follows:

(Equation 1) $$Y_{i,t}=\alpha_{0}+\beta {AirPllt}_{i,t-s}+\gamma X_{it}+\lambda_{i}+\mu_{t}+\varepsilon_{it}$$

Where $Y_{i,t}$ is the hospitalization behaviors and expenditures on day t for district i in Beijing, including the daily clinic visits, daily hospitalization days, daily total hospitalization days, and daily hospitalization expenditures. It is noted that our study period was from January 1, 2015, to December 31, 2019, during which there were no significant public health incidents. For instance, the COVID-19 outbreak, which had a significant impact on public health, began in 2020 and thus did not affect our study period. By selecting an appropriate study period, we aimed to reduce the potential interference from public disease events. AirPllt indicates the daily air pollutant concentrations, measured by PM_2.5_ and PM_10_. Here, we have used a lag of 0 to 7 days (indicated by s) of these air pollutants’ lagged effects on hospitalization behaviors and expenditures.

We included a range of meteorological factors, such as daily average, maximum, and minimum temperatures, wind speed, precipitation, and barometric pressure, to account for any potential confounders that may affect hospitalization behaviors and expenditures. It has been proven that temperature can have a significant impact on human health, leading to various illnesses.^41^^,^^42^ Heavy rainfall can lead to environmental pollution and unsanitary conditions, increasing the risk of infectious diseases.^43^ Barometric pressure affects the supply of oxygen in the body, with low pressure causing shortness of breath and rapid heart rate and high pressure leading to a high incidence of asthma.^44^ Wind speed has also been included as a control variable in the model, as it affects outdoor travel and residence pollution exposure, ultimately impacting human health.

Additionally, we used a district-specific fixed-effect $\lambda_{i}$ to control for unobserved time-invariant determinants of healthcare outcomes across districts. To account for district-common trends in hospitalization behaviors and expenditures during different periods, we applied yearly, monthly, and daily fixed-effects $\mu_{t}$. The random disturbance is represented by $\varepsilon_{it}$.

#### Difference-in-differences model

We conducted a quasi-natural experiment to analyze the impact of cleaner heating policies on different districts. The DID model’s fundamental principle is to ensure that there are no systematic differences between the treated and control groups, except for the implementation of policies. This approach helps to overcome the endogenous influence of the traditional model and provides more accurate estimated results.

We set the DID specifications accordingly:

(Equation 2) $$Y_{i,t}=\alpha_{0}+\beta {Cleanheat\_post}_{i,t}+\gamma X_{it}+\lambda_{i}+\mu_{t}+\varepsilon_{it}$$

Where $Y_{i,t}$ represents hospitalization behaviors and expenditures on day t for district i in Beijing, including daily clinic visits, daily hospitalization days, daily total hospitalization days, and daily hospitalization expenditures. ${Cleanheat\_post}_{i,t}$ is an interaction term obtained by multiplying ${Cleanheat}_{i,t}$ and ${post}_{i,t}$. ${Cleanheat}_{i,t}$ is a dummy variable that indicates whether district i implements cleaner heating policies in time t. Specifically, if district i implements the policy in time t, ${Cleanheat}_{i,t}$ is 1, otherwise 0. We divided 16 districts in Beijing into two groups: one group implemented cleaner heating policies widely with large rural populations (treated group), and the other group did not widely implement cleaner heating policies with small rural populations (control group). ${post}_{i,t}$ is a dummy variable that represents the period when cleaner heating policy was implemented. If the cleaner heating policy was implemented during this period, ${post}_{i,t}$ is 1, otherwise 0. Therefore, the interaction term ${Cleanheat\_post}_{i,t}$ implies the districts that implemented cleaner heating policies and the policy period. In our study, the coefficient β of ${Cleanheat\_post}_{i,t}$ shows the effectiveness of cleaner heating policies on declining hospitalization behaviors and expenditures. We expect the coefficient β to be negative and statistically significant in theory.

It is noted that the DID model allows us to differentiate between the similar characteristics of the treated and control groups, which helps to reduce the interference in the health effects from public disease events and other influencers.

In order to account for any possible external factors, we defined $X_{it}$ in the same way as in the fixed-effect model. Additionally, we incorporated the district-specific fixed-effect $\lambda_{i}$ and time fixed-effect $\mu_{t}$ into the model. Here, the year-fixed effect is used to account for the various unobservable year-specific factors that remained constant across different districts, such as extreme temperature fluctuations. These factors may have an impact on individuals’ health outcomes, and therefore, it is essential to control them. We also included a district-level fixed effect to account for the year-invariant but district-specific characteristics that could potentially cause endogeneity issues. Although the DID model can reduce the differences between each district, the district-fixed effect was necessary to further control for any district-specific characteristics that might impact the estimation outcomes.

#### Data description

We conducted an analysis of daily clinic visits, daily hospitalization days, total daily hospitalization days, and daily hospitalization expenses for all diseases in Beijing. We categorized the health-related variables of all diseases into six categories: respiratory disease, asthma, chronic obstructive pulmonary disease (COPD), stroke, coronary heart disease (CHD), and diabetes. However, due to limited data for each disease, we only provided the results of all diseases rather than each disease at the district level in Beijing on a daily basis. The data collected covers the period between January 1, 2015, and December 31, 2019, and was obtained from the Beijing Municipal Health Commission.

We collected data on daily concentrations of air pollutants to analyze the link between air pollutants and health outcomes. The study focused on two primary air pollutants - particulate matter with an aerodynamic diameter of less than 2.5 or 10 μm (PM_2.5_ and PM_10_, respectively).^45^ These pollutants are highly correlated with the cleaner heating policies and have been identified as the main targets for reduction in several government initiatives. We measured PM_2.5_ and PM_10_ concentrations daily, in sync with health-related variables. Our data source was the China National Urban Air Quality Real-time Publishing Platform. The data on these meteorological variables comes from the China Meteorological Administration.

We measured the health-related impacts in two ways (Table 1): hospitalization days across different sexes and ages and hospitalization-related expenses for males and females, as well as for different age groups. The results of the study showed that, during the period of January 1, 2015, to December 31, 2019, males had an average hospitalization of 1.92 thousand days per district per day across all diseases, while females had an average of 1.81 thousand days. Moreover, people who were more than 60 years old were found to be more susceptible to hospitalization due to disease impacts, with an average of 1.97 thousand days per district per day, higher than age groups of 0-16 (0.12 thousand days) and 17-60 (1.94 thousand days). We also found that hospitalization expenses for males were 3.43 million Yuan per district per day, which was higher than for females (3.10 million Yuan). The comparison of hospitalization expenses among age groups was also provided. Additionally, the daily average temperature was 13°C for each district during the period 2015-2019, while the average concentrations of PM_2.5_ and PM_10_ were 130 and 183 μg/m^3^.
